# A CARE-compliant article successful salvage of bilateral perinatal testicular torsion after delivery out of breech presentation: A case report and review of the literature

**DOI:** 10.1097/MD.0000000000021763

**Published:** 2020-09-04

**Authors:** Vivienne Sommer-Jörgensen, Markus Künzli, Stefan Gerhard Holland-Cunz, Stephanie Gros, Martina Frech

**Affiliations:** Department of Pediatric Surgery, University Children's Hospital Basel UKBB, Basel, Switzerland.

**Keywords:** bilateral orchidopexy, breech presentation, genital birth trauma, neonatal surgical emergency, neonatal testicular torsion

## Abstract

**Rationale:**

Neonatal testicular torsion is a rare condition. There is no consensus about the optimal surgical treatment of neonatal testicular torsions. The salvage rate reported in literature remains poor. Discrimination of the onset of a neonatal testicular torsion seems to have an important impact of the salvage rate. Deliveries out of breech presentation have a risk for neonatal complications including neonatal genital birthtrauma.

**Patient concerns:**

A newborn boy, who was delivered at 40 weeks of gestation out of breech presentation, showed shortly after birth, a painful discoloration and swelling of the scrotum.

**Diagnosis:**

Clinical examination and ultrasound were highly suspicious for testicular torsion.

**Intervention:**

Emergency surgery revealed a bilateral testicular torsion with dark coloration of both testicles. The testicles were detorted and left in situ. A bilateral orchidopexy was performed.

**Outcomes:**

Postoperative ultrasound showed reperfusion of both testicles and the boy was discharged from hospital. Follow-up after 2 months showed normal clinical examination and sonographically persistent perfusion of both testicles. Endocrinological investigations during minipuberty revealed normal hormonal values.

**Lessons:**

We present this rare case in regard to the current literature and recommend close observation of newborns for genital trauma after delivery out of breech position. We encourage surgeons to carefully consider leaving the testicles in situ.

## 1 Introduction

In literature perinatal testicular torsion is most often defined as a vascular event to the testicles during pregnancy or within the first 30 days of life.^[1,2]^ The nomenclature of neonatal testicular torsions is however heterogeneous.^[2]^ Here we use the generic term of neonatal perinatal testicular torsion for events happening during pregnancy (prenatally), birth (perinatally), or within the first 30 days of life (postnatally). Acute onset of scrotal or testicular symptoms is a sign of an acute event, especially in newborns with normal clinical examination of the testicles at birth.^[3]^ The estimated occurrence of neonatal testicular torsions in newborns is 1 in 7500 and about 22% of these cases are of bilateral nature.^[4,5]^ About 25% or even less of neonatal torsions happen peri- or postnatally. The majority of these events occur while still in utero.^[6–8]^ Driver et al^[7]^ reported a rate of neonatal testicular torsions of about 12% of all pediatric cases. There are no guidelines for the management of newborns with testicular torsion and the overall survival rate of the testicles remains poor.^[1,2,8,9]^ Especially in bilateral cases the early recognition of an acute postnatal onset is crucial to prevent anorchia.

## 2 Case report

**Figure 1:**
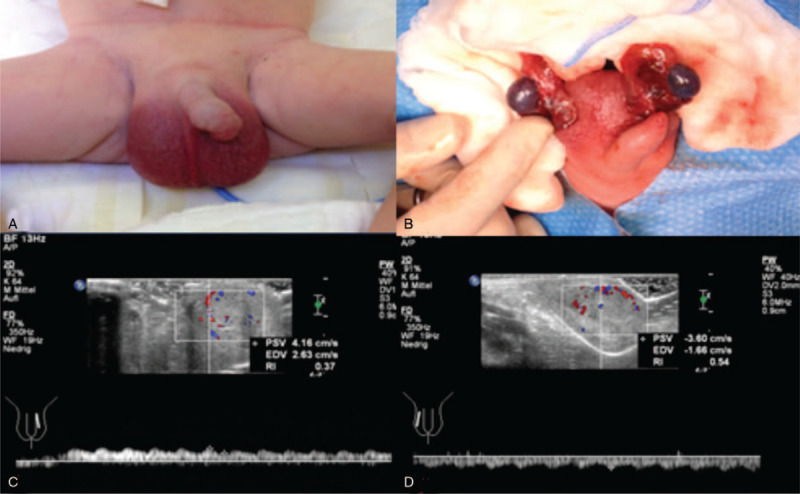
(A) Preoperative situation with scrotal discoloration and swelling, (B) intraoperative finding after detroquing and warm packing. The testicles remain dark with no signs of reperfusion. (C) (right), (D) (left): Postoperative doppler ultrasound of both testicles, showing good perfusion on both sides.

We present the case of a boy who was born out of breech presentation at term (40 weeks of gestation, birth weight 2770 g) in a primary center. Postnatal adaptation was normal (APGAR 9/10/10). Four hours after birth, a discoloration and a significantly painful swelling of the scrotum were noticed. (Fig. 1A) The boy was immediately transferred to our surgical unit. The clinical examination showed the signs mentioned above as well as a hardened testicle in a raised position on the right side. Immediately ultrasound was performed, which showed lack of perfusion and an inhomogeneity of the testicular tissue on both sides. Immediate exploration of the scrotum revealed a bilateral torsion with dark coloration of both testicles. After detorsion and 15 minutes of warm packing only the right testicle seemed to recover slightly, the left testicle remained dark and showed no signs of recovery (Fig. 1B). The decision was made to leave both testicles in situ and bilateral orchidopexy was performed. It was determined to base the decision for a second look operation on the clinical presentation. The postoperative course was uneventful without signs of infection. An ultrasound, performed on the second postoperative day, documented reperfusion of both testicles (Fig. 1 C and D).

At follow-up, after 2, 6, and 12 months, the clinical examination of the external genitalia was unremarkable. Both testicles were located in the scrotum and were of normal size and consistency according to age. In the follow-up ultrasounds, normal homogeneous testicular tissue and perfusion of both testicles were seen. Endocrinological investigation at the age of 2 months during “minipuberty" showed normal hormonal values without any evidence for gonadal dysfunction.

## 3 Discussion/conclusion

### 3.1 Operative management

In the literature there is no consensus about the management of neonatal testicular torsions.^[1]^ Testicular torsion is classified as neonatal during pregnancy and the first 30 days of life^[1]^ regardless of when the event takes place. The operative strategy for patients with prenatal testicular torsions is under controversial discussion.^[1,5,10]^ In most cases an emergent operation would not prevent the loss of the testicles.^[8,9,2]^ But arguments for emergent operation in those patients include the very rare cases of asynchronous acute neonatal testicular torsions and the uncertainty of the onset of the torsion.^[5,11]^ In an acute onset of a perinatal or postnatal testicular torsion, an emergent exploration of the testicles can be crucial to salvage the organs.^[1,2]^

There are only few studies describing exclusively neonatal testicular torsions.^[6,11,3,2]^ Except for the study of Sorensen et al, here is no distinction of the cases regarding the timing of the testicular torsion.^[2]^ The conclusions result from chronic prenatal cases and acute peri- or postnatal cases. There are just 2 reports including only postnatal testicular torsions.^[12,2]^ Sorensen et al describe a series of 10 cases of postnatal testicular torsion, and Granger et al describe a case report of a successfully salvaged testicle.^[12,2]^

There are some clinical indications that might help to determine the time of the torsion. Prenatal testicular torsions might be distinguished by clinical findings during physical examination. Atrophic or fibrotic testicles are assumed to be distorted for a longer period. Nontender or enlarged testicles with induration of the scrotum might be a sign of a more recent event before birth. Prenatally, ultrasound could reveal abnormalities of the testicles, which could be signs of testicular torsion.^[13,11]^ However, a precise timing is not possible in most cases.

Operative management and the survival rate of the testicles are different in acute torsions occurring peri- or postnatally from torsions occurring prenatally. Therefore, a careful discrimination of pre-, peri- or postnatal onset is extremely important. We postulate that neonatal testicular torsions should be classified in prenatal, and peri- or postnatal neonatal testicular torsions to distinguish acute potential reversible events by an urgent scrotal exploration versus chronic nonsalvageable events.

### 3.2 Testicular salvage

The overall prognosis for testicular salvage after neonatal testicular torsions varies between 5% and 40%.^[8,9,2]^ Except for the study of Sorensen et al, the poor percentage of salvage is based on the outcome of all neonatal testicular torsions including chronic prenatal cases.^[2]^ In the only postnatal torsion study, Sorensen et al demonstrate a higher salvage rate of 40% of operated testicles in their patients.^[2]^ In their series 1 resected testicle, which was removed on account of poor intraoperative recovery, proved histologically viable. Considering this additional case the salvage rate may be improved to up to 50%.^[2,10]^ This case demonstrates that patients, which are operated within the ischemic tolerance time, have a chance for testicles salvage even if they present macroscopically in poor condition. Recent studies recommend immediate emergent surgery in all cases if postnatal onset is suspected.^[13,12]^ In these young patients leaving the testicles in situ must be considered, even if they are in a poor condition. This is the only chance to prevent anorchia.

### 3.3 Bilateral testicular torsion

Up to 22% of patients show bilateral occurrence of testicular torsion.^[5]^ They may occur synchronous or metachronous.^[5,11]^ Therefore, exploration of the contralateral side is important and to prevent metachronous onset bilateral fixation of the testicles should be performed. Our case shows that outcome cannot clearly be predicted at time of diagnosis. There is a risk of anorchia in bilateral testicular torsion. In our case, there was no obvious improvement of the testicular perfusion during surgery but the testicles showed full recovery on both sides. Also with regard to the results of Sorensen et al, which showed viable tissue in a removed testicle, we postulate that preservation should be considered even in ischemic testicles without obvious recovery.

### 3.4 Breech position

The presented boy was delivered out of breech presentation and developed first scrotal symptoms a few hours after birth. There are few studies about impressive genital injuries in both boys and girls after delivery out of breech presentation.^[14–16]^ Despite the fact that breech presentation is not a known factor, that promotes neonatal torsion, in this case it seems evident that the mode of delivery may have played a role in the pathogenesis. Delivery out of breech position should raise the suspicion of a testicular injury in case of genital symptoms in a newborn. A careful and close observation of these neonates is essential in detecting testicular injuries early and promoting prompt surgical treatment. Immediate surgical detorsion has an enormous impact on their future life.

## 4 Conclusions

Neonatal bilateral synchronous testicular torsion is a very rare condition. If there is suspicion of an acute onset, an emergent bilateral scrotal exploration is highly recommended. We postulate that neonatal testicular torsions should be classified in prenatal, and peri- or postnatal neonatal testicular torsions to determine the operative management. Considering the catastrophic risk of anorchia in bilateral cases, preservation of the testicles must be considered intraoperatively. We strongly recommend leaving necrotic appearing testicles even without obvious intraoperative recovery primarily in situ. Delivery by breech presentation is a risk factor for neonatal genital birth trauma. We recommend the close observation of these babies.

Written informed consent for the publication of the case was obtained by the parents of the patient.

## Acknowledgment

The authors thank the patient.

## Author contributions

**Data curation:** Markus Künzli.

**Supervision:** Frech Martina.

**Writing – original draft:** Vivienne Sommer-Jörgensen (Bürgin).

**Writing – review & editing:** Holland-Cunz Stefan Gerhard, Gros Stephanie, Frech Martina.
